# Introgression of peanut smut resistance from landraces to elite peanut cultivars (*Arachis hypogaea* L.)

**DOI:** 10.1371/journal.pone.0211920

**Published:** 2019-02-08

**Authors:** Marina Bressano, Alicia N. Massa, Renee S. Arias, Francisco de Blas, Claudio Oddino, Paola C. Faustinelli, Sara Soave, Juan H. Soave, Maria A. Pérez, Victor S. Sobolev, Marshall C. Lamb, Monica Balzarini, Mario I. Buteler, J. Guillermo Seijo

**Affiliations:** 1 Facultad de Ciencias Agropecuarias-Universidad Nacional de Córdoba, Córdoba, Argentina; 2 USDA-ARS-National Peanut Research Laboratory (NPRL), Dawson, GA, United States of America; 3 Instituto Multidisciplinario de Biología Vegetal-(IMBIV-CONICET-UNC), Córdoba, Argentina; 4 Criadero El Carmen, General Cabrera, Córdoba, Argentina; 5 Instituto de Botánica del Nordeste (IBONE, CONICET-UNNE) y Facultad de Ciencias Exactas y Naturales, Universidad Nacional del Nordeste, Corrientes, Argentina; Osmania University, INDIA

## Abstract

Smut disease caused by the fungal pathogen *Thecaphora frezii* Carranza & Lindquist is threatening the peanut production in Argentina. Fungicides commonly used in the peanut crop have shown little or no effect controlling the disease, making it a priority to obtain peanut varieties resistant to smut. In this study, recombinant inbred lines (RILs) were developed from three crosses between three susceptible peanut elite cultivars (*Arachis hypogaea* L. subsp. *hypogaea*) and two resistant landraces (*Arachis hypogaea* L. subsp. *fastigiata* Waldron). Parents and RILs were evaluated under high inoculum pressure (12000 teliospores g^-1^ of soil) over three years. Disease resistance parameters showed a broad range of variation with incidence mean values ranging from 1.0 to 35.0% and disease severity index ranging from 0.01 to 0.30. Average heritability (*h*^*2*^) estimates of 0.61 to 0.73 indicated that resistance in the RILs was heritable, with several lines (4 to 7 from each cross) showing a high degree of resistance and stability over three years. Evidence of genetic transfer between genetically distinguishable germplasm (introgression in a broad sense) was further supported by simple-sequence repeats (SSRs) and Insertion/Deletion (InDel) marker genotyping. This is the first report of smut genetic resistance identified in peanut landraces and its introgression into elite peanut cultivars.

## Introduction

Smut disease is threatening the peanut production in Argentina [1]. Though Argentina produces only 1 Mt of the 38 Mt generated worldwide [2], it exports 80% of the production for approximately $ 800 million U.S. dollars, making it the first peanut exporter in the world (Camara Argentina del Mani, available from: www.camaradelmani.org.ar). Peanut smut disease, which is caused by the fungal pathogen *Thecaphora frezii* [3, 4], has been observed in 100% of the peanut production area of Argentina, 350,000 ha [5–7]. In highly infested areas, the disease incidence can reach up to 52% accompanied by yield losses of 35% [7]. *Thecaphora frezii* invades the pegs as they enter the ground, then consuming the peanut seeds and leaving behind a mass of dark-brown teliospores [4]. The teliospores accumulate in soil, building up inoculum with each cropping season [8]. Fungicides commonly used in the peanut crop have shown little or no effect controlling peanut smut [9–11], making it an utmost priority to obtain peanut varieties resistant to this disease.

The narrow genetic base of cultivated peanut has been well documented [12–14]. Therefore, wild peanuts have received much consideration as sources of genetic variation and potential disease resistance [15–19]. Being the cultivated peanut (*A*. *hypogaea*) an allotetraploid [20], the incorporation of genetic material from wild diploid species requires generating synthetic amphidiploids [21, 22]. On the contrary, peanut landraces are an alternative source of genetic variability. These germplasms are valuable resources from the primary gene pool that can be immediately incorporated into commercial cultivars [23, 24].

As part of a large breeding program from Criadero El Carmen to develop peanut varieties with resistance to smut disease, hundreds of accessions including landraces, advanced breeding lines, and elite peanut varieties were previously screened. Two of those germplasms, which were identified as resistant, were later crossed with susceptible commercial peanut cultivars. Here we report a multi-year phenotyping of three crosses between susceptible and resistant lines, as well as the genetic fingerprinting of parents and progeny of these crosses using simple-sequence repeats (SSRs) and Insertion/Deletion (InDel) markers.

## Materials and methods

### Plant material

Three recombinant inbred lines (RILs) were developed from crosses between three susceptible peanut cultivars, Granoleico, Guasu, and I1014 and two resistant germplasms, I0322 and I0349 (Table 1). All parental lines used in the crosses are tetraploid (2*n*  =  4*x*  =  40). The line I0322 was selected from a landrace of *Arachis hypogaea* L subsp. *fastigiata* Waldron var. *fastigiata* (Waldron) Krapov. & W. C. Greg) introduced from Bolivia [25]. The line I0349 originated from a genetically heterogeneous germplasm identified as *Arachis hypogaea* L, resembling var. *fastigiata*, although different from the *fastigiata* type (G. Seijo, personal communication). Further taxonomic characterization is needed to elucidate the genetic identity of this germplasm. The cross I0322×Guasu (JS31411) was performed during the 2010–2011 growing season, while I0349×I1014 (JS35112) and I0349×Granoleico (JS34212) were made during 2011–2012. The initial size of the F_2_ population from each cross was reduced to around 20% of the progeny by keeping the most resistant lines and advancing them to F_6_ and F_7_ by single seed descent. Accordingly, the final number of lines in each of the crosses ranged from 16 to 19 (Table 1).

**Table 1:** Description of crosses and progeny.

**Cross**	**Parental lines** **(Female × Male)**	**Progeny** **(N**^**o**^ **of RILs)**
**JS31411**	GUASÚ **×** I0322	16
**JS34212**	GRANOLEICO **×** I0349	19
**JS35112**	I1014 **×** I0349	18

### Disease assessment

The F_5_, F_6_ and F_7_ RIL generations of JS31411 and the F_4_, F_5_, F_6_ RIL generations of JS35112 and JS34212 were evaluated at the Criadero El Carmen experimental farm located in General Cabrera, Cordoba, Argentina (32°49'46"S, 63°52'12"W). The evaluations were performed during the growing seasons of 2014–15, 2015–16, and 2016–17. Single-row plots (2.5 m long) were arranged in a randomized complete block design with two replicates. Plots were planted in infested soils containing an average of 12000 of *T*. *frezii* teliospores g^-1^ of soil. This inoculum density is three times higher than the average concentration (18–4400 teliospores g^-1^ of soil) present in naturally smut-infested fields of the peanut-growing area of Argentina [8]. The presence of *T*. *frezii* in soil was assessed by teliospores counting according to the method of Marinelli et al. [4]. Standard agronomic practices, with no irrigation, were applied to control weeds and other peanut diseases. The entire plots were harvested at physiological maturity determined by the nursery standards for breeding purposes. One hundred randomly selected mature pods per plot were manually opened and visually assessed to score disease incidence and disease index (DI) as follows:

$$Disease\;Incidence\;(\%)=\left(\frac{n{}^{\circ}\;of\;infected\;pods}{n{}^{\circ}\;of\;total\;pods}\right)\times 100$$ (1)

$$Disease\;Index=\left(\frac{\Sigma (severity\;class\times number\;of\;infected\;pods)}{n{}^{\circ}\;of\;total\;pods\times maximum\;severity\;class}\right)$$ (2)

**Fig 1:**
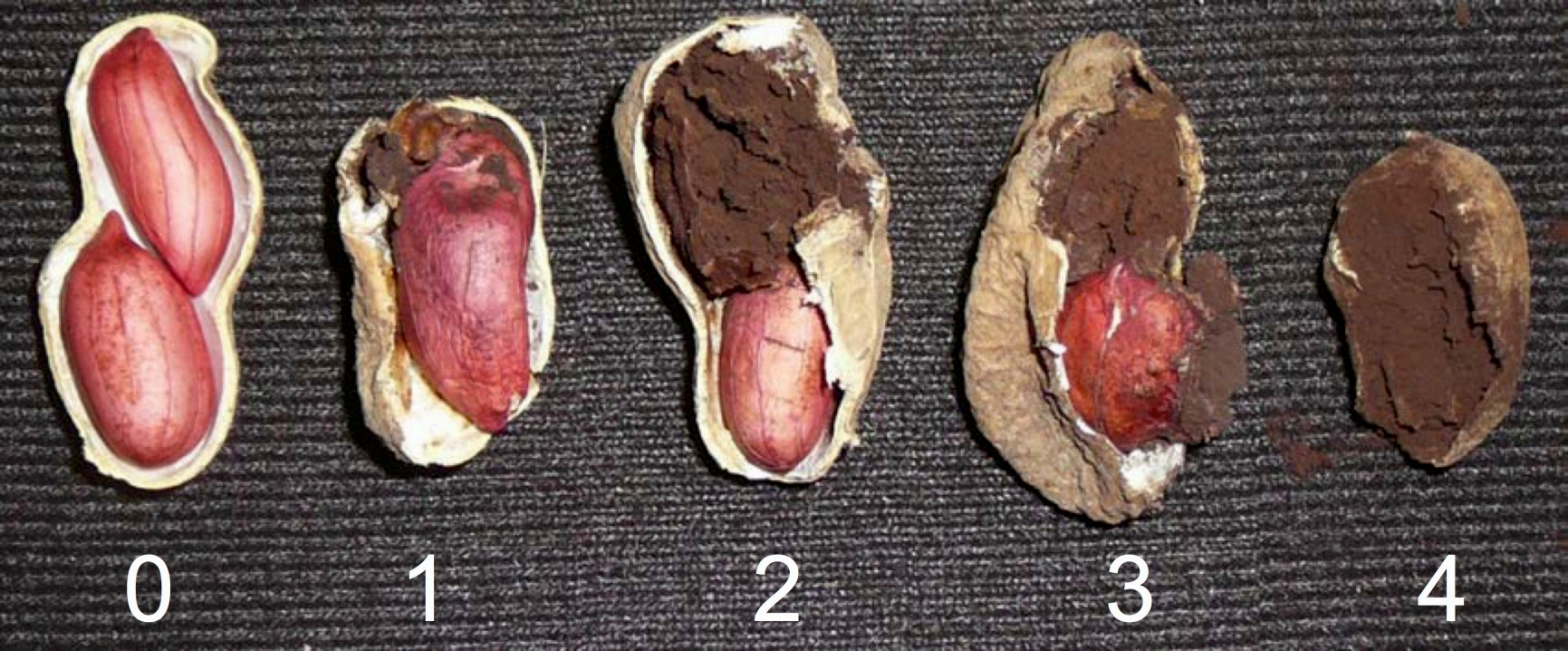
Peanut smut severity scale. Classes on a 0 to 4 scale, where 0 = healthy pods, 1 = normal pod with a small sorus in single kernel, 2 = deformed or normal pod with half of the kernels affected, 3 = deformed pod and one completely smutted kernel, and 4 = deformed pod with all kernels completely smutted [1].

Disease index estimates were based on McKinney infection rating formula [26]. Severity classes were determined on a 0 to 4 scale as described in [1], where 0 = healthy pods; 1 = normal pod with a small sorus in single kernel; 2 = deformed or normal pod with half of the kernels affected; 3 = deformed pod and one completely smutted kernel; and 4 = deformed pod with all kernels completely smutted (Fig 1).

Meteorological conditions for the three seasons of phenotyping were extracted for the department Juarez Celman, Cordoba, Argentina, from the Red de Estaciones Meteorologicas, summarized every year by Bolsa de Cereales de Cordoba [5, 27–29]. Analysis of Variance on Ranks was performed for average maximum temperatures and rain during the three cropping seasons; mean values were compared by Tukey’s or Dunn’s tests using the statistical package Sigma Plot v. 12.5 (Systat Software Inc., San Jose, CA).

### Genotypic variability characterized by SSR and InDel markers

Advanced lines from the three peanut crosses between resistant and susceptible parents (Table 1) were multiplied in a growth chamber and all individuals (47 progeny and 5 parents) were fingerprinted with SSRs and InDel markers. DNA was extracted from young leaves using DNeasy PowerPlant Pro Kit (Qiagen) and CTAB method [30]. The quantity and quality of DNA was assessed by electrophoresis in 1% agarose gels using phage *lambda/HindIII* marker (Pb-L Productos Bio-Lógicos, Argentina) for quality control.

The parents of the three populations were screened with 376 molecular markers: 288 SSRs [31], 12 insertion/deletion (InDel) markers [32], and 73 SSRs obtained from the literature for peanut and related species [14, 33–37]. The complete list of 376 primer sets was previously reported (Arias et al., 2018). From these markers, 94 were selected to screen the progenies of the three crosses. Forward primers were 5’ tailed with the sequence 5’-CAGTTTTCCCAGTCACGAC-3’ (Waldbieser et al., 2003) and reverse primers were tailed at the 5’ end with the sequence 5’-GTTT-3’ (Brownstein et al., 1996). Primer 5’-CAGTTTTCCCAGTCACGAC-3’ labeled with 6-carboxy-X-rhodamine (ROX) (IDT-Technologies, Coralville, IA) was used for amplification of 10-ng DNA in a 5 μL reaction using Titanium Taq DNA Polymerase (Clontech, Mountain View, CA) as reported before [38]. Fluorescently-labeled PCR fragments were analyzed by capillary electrophoresis on an ABI 3730XL DNA Analyzer (Applied Biosystems, Foster City, CA) and data were processed using Gene Mapper software 4.0 (Applied Biosystems, Foster City, CA). PCR amplicon scoring was recorded as allele size in base pairs (bp) allele size and converted to binary data as zeroes (absence) and ones (presence). Given the complexity of the allotetraploid genome of cultivated peanut, where similar size amplicons could correspond to different sub-genomes, each amplicon observed in the molecular marker data was analyzed as a dominant marker. Thus, no true heterozygosity was recorded. The number of alleles per locus and allele size range (bp) were determined for each primer set. Polymorphic information content (PIC) for each marker was calculated according to Botstein et al. [39]. For each of the crosses, 3D-Principal Coordinate Analysis (PCoA) [40] was performed using NTSYSpc v. 2.2, [41] (Exeter Software, Setauket, NY). For each progeny, allele contribution from each parent was calculated for alleles present in only one of the parents, and the values were expressed as percentage. DNA sequences containing the SSR and InDel markers were mapped to the genome assemblies of *A*. *duranensis* and *A*. *ipaënsis* [42] using BLAST [43].

### Phenotypic statistical analysis

Incidence and DI phenotypic values were square root transformed and subjected to statistical analysis. Trait heritability was estimated based on parent-offspring regression/correlation analysis. This method does not require the assumption of normality as in the analysis of variance and in self-pollinated species such as peanut the regression coefficient is equal to the narrow sense heritability (*h*^*2*^) of a trait [44]. Given that parent (*e*.*g*. F_5_) and progeny (*e*.*g*. F_6_) generations were evaluated in different environments (different years), the correlation (Pearson’s) rather than the regression coefficient was used in order to decrease the potential environmental effects.

Narrow sense heritability was calculated according to the equation:

$$h^{2}=r_{FxFy}=\frac{Cov_{FxFy}}{{\left(V_{Fx}V_{Fy}\right)}^{1/2}}$$

where *r* is the Pearson’s correlation, *Cov* is the covariance, and *V*_*Fx*_ and *V*_*Fy*_ are the variance in the parental (*e*.*g*. F_5_) and progeny (*e*.*g*. F_6_) generations, respectively.

Correlation analyses were further performed to determine the relationships between disease measurements (incidence and DI). Correlation coefficients were calculated and plotted using the Corrplot R package [45]. Tests for association between trait and SSR/InDel markers were conducted using the simple linear regression model (lm), *p*-values were adjusted for multiple testing using the Bonferroni correction, and means were compared using the Tukey test. All statistical analyses were performed in R software [46]. To test the significance of smut disease resistance/susceptibility variability, incidence mean values were subjected to analysis of variance and then compared by the Scott-Knott clustering algorithm with a α value of 0.05, using the ScottKnott R package [47].

## Results

### Phenotypic statistical analysis

**Fig 2:**
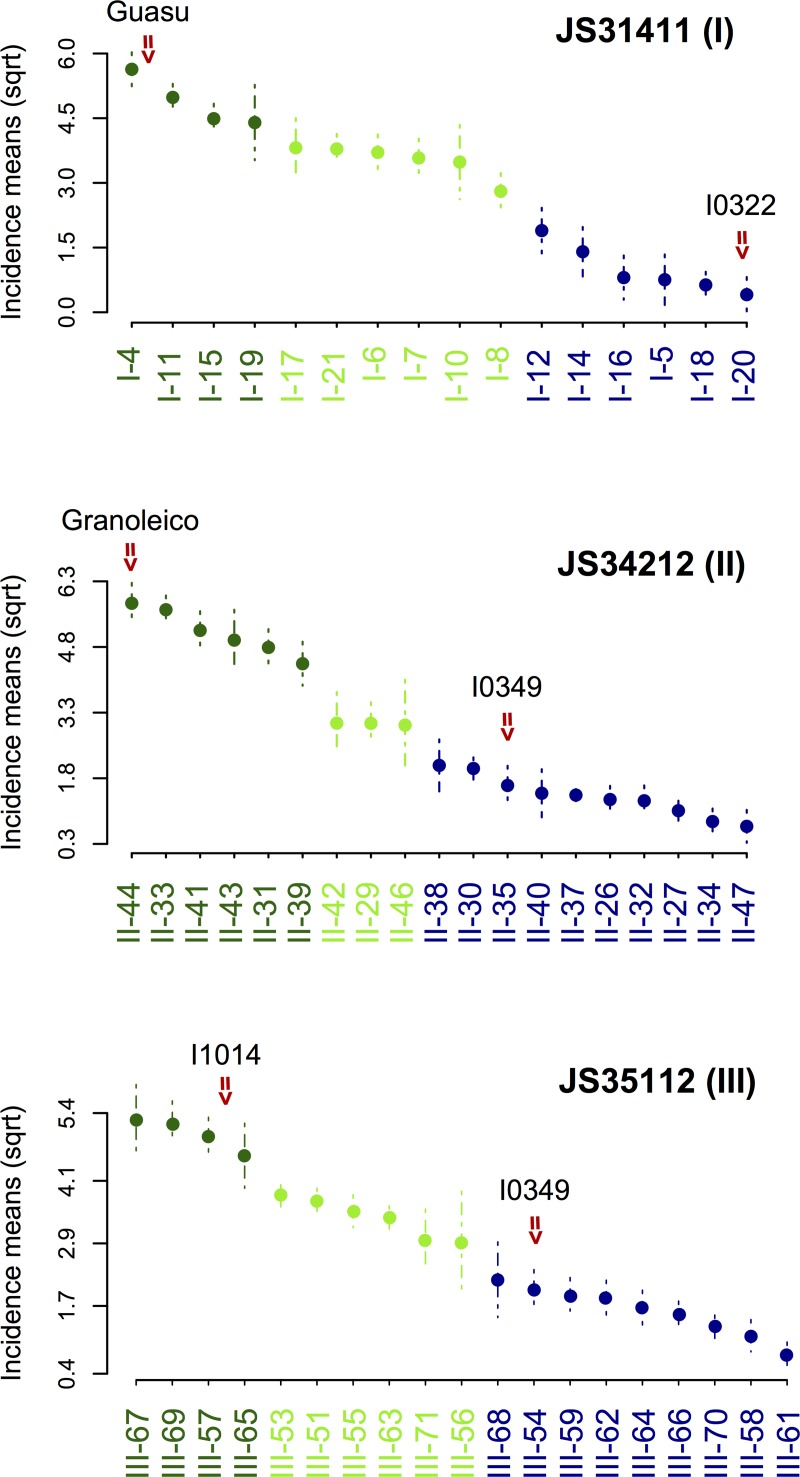
Genotype groups for incidence mean values as defined by the Scott-Knott algorithm (*α* = 0.05).

Screening in an environment with high inoculum pressure allowed the development of high intensity smut symptoms as well as the discrimination between levels of resistance. Disease resistance measurements within and across generations exhibited a broad range of phenotypic variation with incidence mean values ranging from 1.0 to 35.0% and disease severity index ranging from 0.01 to 0.30. Of the two resistant parental lines, I0322 exhibited the highest levels of resistance with a mean incidence value of 0.43% and a DI mean value close to zero. Among the susceptible parents, Granoleico showed the highest disease incidence (44.5%) and DI (0.30) scores (Table 2, S1 Table). Recombinant inbred lines derived from crosses JS34212 (II) and JS35112 (III) showed transgressive phenotypes with incidence and disease index values lower than the mean-parent values of the common resistant parent I0349. No transgressive segregants for smut resistance were observed in RILs derived from the cross JS31411 (I) as I0322 is highly resistant (nearly immune) (Fig 2). While it was beyond the scope of this paper to present the analysis of the crosses based on agronomic performance, it is worth noting that two of the resistant lines, one from cross JS31411 (line I-14) and one from cross JS35112 (line III-61) showed favorable agronomic characteristics (J. Soave personal communication).

**Table 2:** Smut disease incidence and disease index mean, standard error (SE), and range for the parental lines (P1, P2) and generations of RILs from the three peanut corsses: JS31411 (I), JS34212 (II), and JS35112 (III).

Cross	Genotypes	Incidence (%)		Disease Index	
		Mean ± SE	Range	Mean ± SE	Range
JS31411 (I)	P1 (I0322)	0.43 ± 0.31	2.90	0.00 ± 0.00	0.03
	P2 (Guasu)	27.80 ± 4.50	42.40	0.18 ± 0.03	0.27
	F_5_-RILs	12.80 ± 2.00	42.70	0.09 ± 0.02	0.33
	F_6_-RILs	13.36 ± 2.94	65.20	0.10 ± 0.02	0.51
	F_7_-RILs	11.37 ± 1.98	39.00	0.07 ± 0.01	0.25
JS34212 (II)	P1 (I0349)	3.58 ± 1.11	8.80	0.02 ± 0.01	0.05
	P2 (Granoleico)	44.5 ± 3.69	34.30	0.30 ± 0.03	0.37
	F_4_-RILs	9.95 ± 2.09	41.30	0.07 ± 0.01	0.27
	F_5_-RILs	15.78 ± 2.57	56.60	0.12 ± 0.02	0.48
	F_6_-RILs	10.82 ± 2.00	49.00	0.07 ± 0.01	0.32
JS35112 (III)	P1 (I0349)	3.58 ± 1.11	8.80	0.02 ± 0.01	0.05
	P2 (I1014)	17.98 ± 2.17	9.80	0.11 ± 0.01	0.05
	F_4_-RILs	8.41 ± 1.57	41.18	0.06 ± 0.01	0.34
	F_5_-RILs	8.47 ± 1.63	34.40	0.06 ± 0.01	0.27
	F_6_-RILs	9.06 ± 1.50	32.10	0.05 ± 0.01	0.20

**Fig 3:**
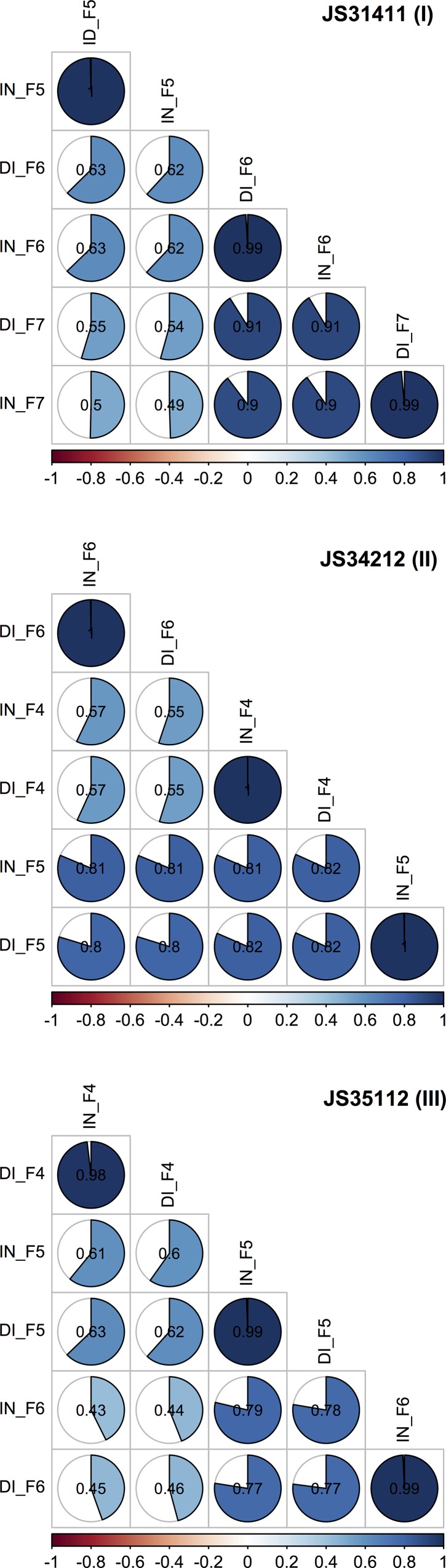
Correlogram showing the correlations (*P*-value ≤ 0.01) between traits and generation of RILs. Pearson’s correlation coefficients are indicated in the cells.

Correlation coefficients were calculated both, at the trait and generation levels. At the trait level, a strong correlation (≥ 0.80, *P* ≤ 0.01) was observed between incidence and DI measurements for each generation of RIL (Fig 3). At the generation level, correlation coefficients for the same trait in different generations corresponded to the narrow heritability (*h*^2^) of the trait. Correlation coefficients ranged from 0.43 (IN, JS35112) to 0.91 (DI, JS31411) indicating moderate (≥ 0.40) to strong (≥ 0.80) heritability. Higher correlations between successive generations, particularly between more advanced generations, suggest parent/offspring consistency in the response to the disease (Fig 3).

Significant phenotypic differences among genotype means (averaged across generations) were clustered into groups by the Scott-Knott algorithm (*P* ≤ 0.05). Fig 2 shows the results based on disease incidence values for all three crosses. For cross JS3411 (I), among the 16 RILs, 6 were classified as resistant (*i*.*e*. incidence means were not significantly different from that of the resistant parent I0322), six as intermediate, and four as susceptible (*i*.*e*. incidence means were not significantly different from that of the susceptible parent Guasu). Similar patterns of grouping were observed in cross JS34212 (II). For cross JS351112 (III), RILs with intermediate values were split into two groups, based mainly on standard error differences. RILs III-71 and III-56 exhibited higher standard errors compared to the group of RILs with similar intermediate incidence values (i.e., III-51, III-53, III55, III-63).

### Weather during phenotyping seasons

Meteorological data, including monthly rain and the averages of maximum/minimum temperatures during the peanut cropping seasons (December to May) 2014/15, 2015/16 and 2016/17 in the department Juarez Celman, Cordoba, are plotted in S1 Fig. Overall, 2014/15 was warmer (max average temperatures for April and May, 28 and 22°C, respectively) for the last two months of the crop than 2015/16 or 2016/17 (23±1–17±1°C) (p ≤ 0.05). The beginning of the cropping season (Jan/Feb/Mar) 2016/17 received significantly (p ≤ 0.05) less rain (91 mm) than seasons 2014/15 and 2015/16 (138–141 mm, respectively).

### Molecular markers

From a set of 94 SSR/InDel markers that were selected to genotype the parents and RILs of the three crosses, 37 (39%) showed non-transferability, that is, did not amplify any of the parents. After an initial filtering to remove markers that failed to amplify in one or more of the parents, and/or markers with low-quality amplification, 52 markers (312 alleles) were available for further analyses (S2 Table). A list of primer sets, including number of alleles per locus and allele size range is shown in Table 3. The number of markers per crosses ranged from 47 to 52, while the number of alleles ranged from 180 to 226 (Table 4). Alleles present in the progeny and in only one of the parents were referred as “parent specific alleles” (PSA) (Table 4, S3 Table).

**Table 3:** Molecular markers used in genotyping and analysis of three peanut crosses between smut-resistant landraces and susceptible elite cultivars. Crosses were JS31411 (I), JS34212 (II) and JS35112 (III). Numbers in parentheses indicate the references for markers: (1) [37], (2) [36], (3) [14], (4) [48], (5) [32], the rest of the markers were recently reported [31].

Marker		Sequence 5' → 3'	No. of alleles	Amplicon size (bp)	PIC
NPRL_A0A9P	F:	CCTAGTTGCTTCCGTAACCGACAT	4	140–361	0.619
	R:	TCAGCCTAAGCACACACCAAGT			
NPRL_AAJZM	F:	CTGGCTGCCTTATAATCACCACAT	1	140	0.000
	R:	TCAGCAGAAGAATCACCACTACTCC			
NPRL_ABCLW	F:	CCAACTTGAACATCTTCTTGTCCA	1	138	0.000
	R:	TCGTTCTTTCAGGTTTTTCACCAT			
NPRL_AKH02	F:	CGTGTTCTTGCAGCAGTATG	15	108–412	0.551
	R:	ACACTACCACCACACTAGAC			
NPRL_BULCQ	F:	GCTCGCATCG TTGAGATCAAC	7	95–487	0.764
	R:	TTGGCTTCCAAGGTCTTC			
NPRL_cont00136a	F:	TAACCCTACGACATCTGCATCTCA	6	139–352	0.142
	R:	CTGCTACTCCATTCCGTCATTCTT			
NPRL_cont00201a	F:	TGACAACGGTTGAAAAGGAGATTT	5	160–463	0.666
	R:	CCTTCACAACCTTACATTCCCAAG			
NPRL_cont00236a	F:	TCTACCCAACAACCCACCTCATAG	5	176–393	0.706
	R:	ATTCATGTAGCCGACCCCACTTA			
NPRL_cont00250a	F:	TGTTGCTGAGAAGATGATGGAAAA	11	154–487	0.699
	R:	CATGGGAGAATCCTATGGAAACAC			
NPRL_cont00266a	F:	TTTTTCCTTCTCCTACCCCTCATC	6	138–451	0.722
	R:	CACTTGTCAAAGAAAAGAGAAAGCG			
NPRL_cont00289a	F:	AATTCGTCCTACTCTCACAGTCCG	2	153–229	0.500
	R:	ATTGCTGATGACAATGACGATGAC			
NPRL_cont00381a	F:	CAACCATCATCCAACACTACCAAT	9	148–433	0.518
	R:	TCAGCAGCAATAAACAGTAAACAAGA			
NPRL_cont00405a	F:	CCACTATCATTTCCCATCCACAAT	2	160–179	0.639
	R:	CAGCAGCAATATCTCAGCACAATC			
NPRL_cont00460a	F:	CTAATGGGTGCAGGGATGTAAAAT	4	129–372	0.595
	R:	TATTGAGGGATTGGTCAAGGTGTT			
NPRL_cont00551a	F:	CCTTCACCTGGAGCTAGTGAAATC	2	145–293	0.074
	R:	GTTGAGGGCTGTTCTTGATGAGAT			
NPRL_cont00644a	F:	AGCTCCGAGGAGAAGAAGCTAAAC	3	125–277	0.145
	R:	ATTTGGCTTCGATCTGAAGATTTG			
NPRL_cont00661a	F:	TTGTCATCTTTGACATCACCGTTT	5	171–344	0.534
	R:	CCACCTCTATCATCATCATGGCT			
NPRL_cont00761a	F:	AGTTCCAAGTAACCACATCCCTCA	6	233–346	0.700
	R:	CCTGGTCATATCATCCAAACACAA			
NPRL_cont00778a	F:	CCATTATCTTCAAAACGAATCCAAA	4	148–271	0.686
	R:	TTGTTTCGTTCTTCGTTCTTCTCC			
NPRL_cont00816a	F:	TCGACTATGAGAAGAACGAAGAGAAA	3	99–340	0.426
	R:	AAGAACCACATTCTGAAGGTCCAC			
NPRL_cont00843a	F:	TCAGCAACTCCAAGACCTTCTCTT	7	101–371	0.691
	R:	AAAAGAGTGCGAGAAGTGAAATGG			
NPRL_cont00873a	F:	TCACTAACCGCATCTTCTTTGTCA	4	158–494	0.500
	R:	TAGAATGTGTTTGTGAAGGTTGCG			
NPRL_cont00921a	F:	CCTCATGCCATAAAGCAAAGGTTA	9	124–422	0.609
	R:	CGTGCTTTGTAATGCCATATTTGA			
NPRL_cont01163a	F:	AGAGCAGAATGCTTGGTCCATA	6	105–456	0.683
	R:	TTACAACCTTTTATCAGTCTTCACTGC			
NPRL_cont01192a	F:	GTTGCATATGTGGATGGAGAACAA	6	129–390	0.500
	R:	GCGTTGACGAAGTCAGACTAAGGT			
NPRL_cont01277a	F:	ACAGCATGCCAGAGAAACCTAATC	12	115–495	0.782
	R:	GATGGGCTTAGCAACCATATTGAC			
NPRL_cont01294a	F:	GGAAGGATTAAGCATCATCAACCA	11	114–478	0.751
	R:	CAATACAACCTTTTGGAGTTCGCT			
NPRL_cont01491a	F:	CTAGGTGGTCGACGGTGGTG	8	110–479	0.544
	R:	CCCCTTTCTTCTTCCTCTTCTCTG			
NPRL_cont01513a	F:	GAGAGAAAAAGGTTCCTCCCTAAGC	11	123–495	0.653
	R:	CGCTTGATTTAGCTTGGAGTTTCT			
NPRL_cont01577a	F:	CTGAGAAGAGGATACGCGAGTGAG	1	242	0.000
	R:	TCATCTGCATCATCTTTTCCCTCT			
NPRL_cont01653a	F:	GAAAGAGGAAGAAGAACGTGTAGCA	4	123–477	0.667
	R:	ATTCAAGGTACGTTTCTTGCCTTC			
NPRL_cont01725a	F:	TTGTTGTCAACTCTAAGCAAGACAAA	8	114–460	0.641
	R:	GCACAACTTGAATTTAGGTTTCCTC			
NPRL_cont02046a	F:	AGCAACAACTCAACCTCAGATGAA	5	182–340	0.675
	R:	TGGTTCTTTGTTGTCAATTCTTGG			
NPRL_cont02343a	F:	GTTTCTAGTGGTTGCGATGTTTCT	6	182–340	0.620
	R:	CAACAACCTTGAAGCTCCTACTCT			
NPRL_cont02426a	F:	TAATCTCAGCCGTCCGATTTAGAC	7	127–366	0.667
	R:	CTACTCACACAGCAACGAACAGC			
NPRL_cont02651a	F:	AATGAAGGAAGGGAAGGAAGGAAG	12	111–466	0.560
	R:	AAAGAAGAAAGGGGTCCTTGGATT			
RM14E11 (1)	F:	CCATCCAATCAGCAATCACTAA	5	118–414	0.659
	R:	GAAGAAGAAGAGGAATGCCAGA			
RN31E06 (1)	F:	AGGGACAGCATTTCCAAGATGA	4	127–364	0.652
	R:	AGGCGGCCGACATGTTTT			
Ah1TC11A02 (2)	F:	AATCGGAATGGCAAGAGACA	7	136–429	0.738
	R:	AGAGCAAAGGGCGAATCTATG			
Ah1TC1D02 (2)	F:	GATCCAAAATCTCGCCTTGA	5	135–423	0.355
	R:	GCTGCTCTGCACAACAAGAA			
Ah1TC2D06 (2)	F:	AGGGGGAGTCAAAGGAAAGA	5	101–276	0.690
	R:	TCACGATCCCTTCTCCTTCA			
Ah1TC3H02 (2)	F:	CTCTCCGCCATCCATGTAAT	10	102–320	0.603
	R:	ATGGTGAGCTCGACGCTAGT			
Ah1TC6E01 (2)	F:	CTCCCTCGCTTCCTCTTTCT	7	142–427	0.766
	R:	ACGCATTAACCACACACCAA			
Ah1TC7E04 (2)	F:	GAAGGACCCCATCTATTCAAA	7	283–439	0.709
	R:	TCCGATTTCTCTCTCTCTCTCTC			
Ah1TC9F04 (2)	F:	CCTAAACAACGACAAACACTCA	13	133–499	0.623
	R:	AAGCACAACACAGAACCCTAAA			
AS1RI1F06 (2)	F:	TGTCTCTCTTCCTTTCCTTGCT	3	103–408	0.239
	R:	CCTTTTGCTTCTTTGCTTCC			
AS1RN3E10 (2)	F:	TAGAAGAAGGAGAGGGTGAGAA	4	261–416	0.666
	R:	CTAAGATGGTGGTGGGAATTA			
gi-30419832 (2)	F:	GCCACTTTATTCTAAGCACTCC	3	205–360	0.142
	R:	AAGAGACCACACGCTCACA			
Ah-202 (3)	F:	AATTGAGGGTGCTCTTCAGCC	4	208–307	0.525
	R:	ATGAGGCTGGGGTTGAGAAGAT			
pPGPseq2E6R (4)	F:	CCTGGGCTGGGGTATTATTT	2	119–137	0.429
	R:	GCACACCATGGCTCAGTTATT			
Indel-016 (5)	F:	TCCTCATCAGGAACTGGGATA	5	176–348	0.498
	R:	TGCAGCAATAGGACTTCTGG			
Indel-030 (5)	F:	TTGAAGGCAGAGGAGGTAGC	4	125–266	0.146
	R:	GAAAGGAACATTGAACTAAATTTTGC			
Indel-046 (5)	F:	TGAACTCGAGCGAACATCAC	6	106–484	0.344
	R:	TTTGTGCTTTGGCACCATTA			

**Table 4:** Summary of molecular marker analysis for each of the crosses. P1: susceptible parent; P2: resistant parent. Parent specific alleles (PSA) are alleles present in only one of the parents. Shared alleles: are alleles present in both parents and in all their progeny. PSA in progeny: is the average number of alleles present in each individual progeny that originated either from parent P1 or P2 (exclusively).

Cross	Loci	Parent specific alleles (PSA)	Total alleles	Shared alleles	Average PSA in the RILs	Chromosomal location for the alleles of P2 based on BLAST to the *A*. *duranensis* (A) and *A*. *ipaënsis* (B) genome assemblies
JS31411 (I)	52		180	68		
P1 (Guasu)		46			16 (10 to 21)	
P2 (I0322)		41			18 (14 to 20)	A01/B03, A04, A05/B05, A08, A10, A10/B10, B07, B08, B10
					34 (26 to 40)	
JS34212 (II)	45		217	22		
P1 (Granol.)		96			61 (57 to 64)	
P2 (I0349)		88			6 (3 to 10)	A06, A10, B07, B08, B10
					67 (63 to 72)	
JS35112 (III)	47		226	25		
P1 (I1014)		102			71 (66 to 76)	
P2 (I0349)		89				A02, A02/B02 A04, A06, A08, A09/B09, A10, B08, B10
					75 (71 to 80)	

#### Cluster analysis

**Fig 4:**
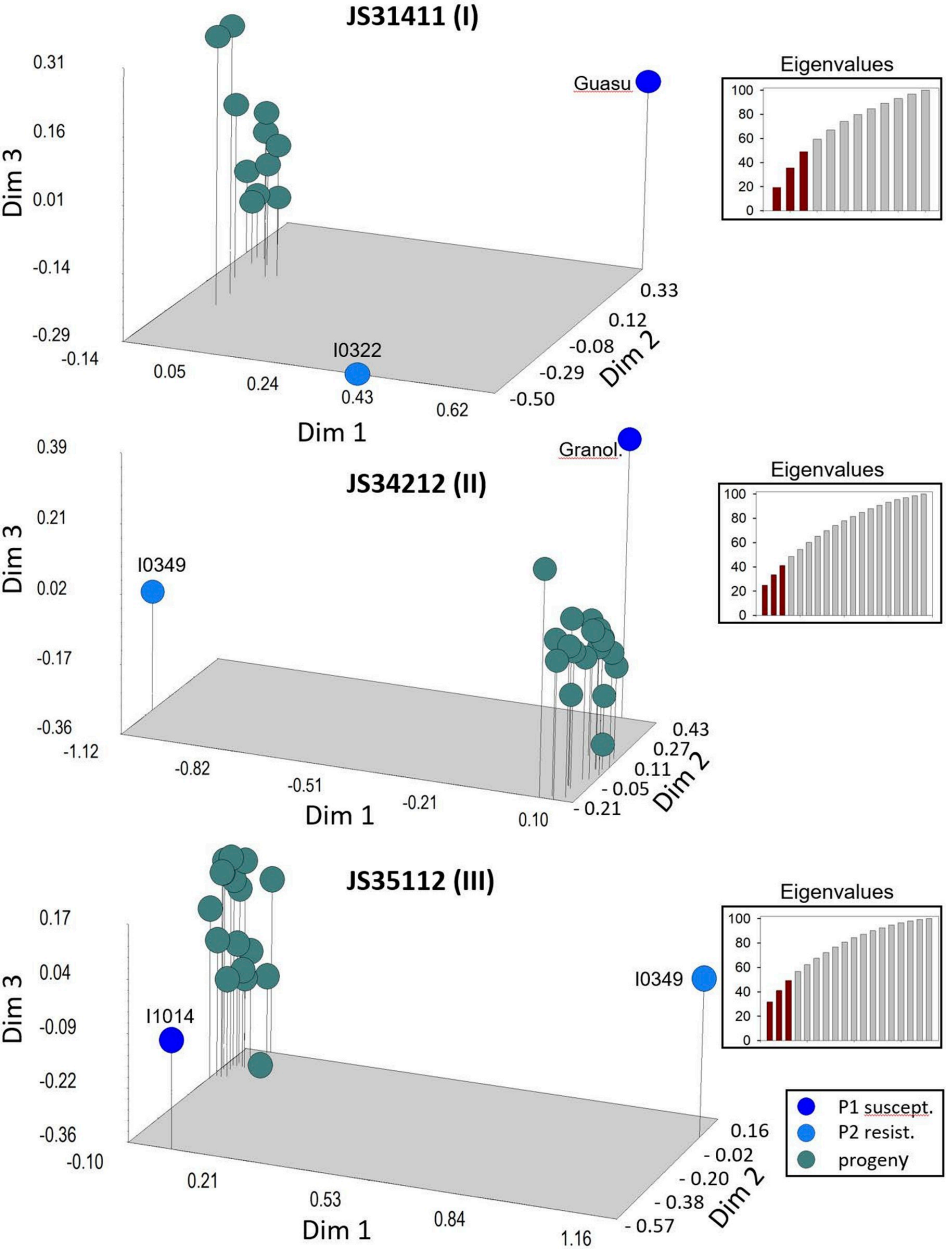
3D-Principal Coordinate Analysis (3D-PCoA) of genetic distances calculated for parents and progeny of three peanut crosses: JS31411 (I), JS34212 (II) and JS35112 (III) at 52, 45 and 47 loci, respectively. Total number of alleles observed: 180, 217 and 226 for cross JS31411 (I), JS34212 (II) and JS35112 (III), respectively.

The 3D principal-coordinate analysis (3D-PCoA) for each of the three crosses showed each progeny as a single cluster with no evidence of outliers and clearly separated from the parental genotypes (P1, P2, Fig 4). In crosses JS34212 (II) and JS35112 (III) the progenies were closer to the susceptible parent (P1) than to the resistant one (P2, Fig 4, S2 Fig). The first coordinate, Dim-1, in all three crosses clearly separated the progeny from the resistant parents, and in crosses JS34212 (II) and JS35112 (III) Dim-1 also separated the resistant from the susceptible parents. In cross JS31411 (I), this separation was more effective by the second coordinate, Dim-2. The percentage of genetic variation explained by Dim-1 was 19.2, 24.9 and 31.2, for crosses I, II and III, respectively. The percentage of the genetic variation explained by the first three coordinates combined (Dim-1, Dim-2 and Dim-3) for crosses JS31411 (I), JS34212 (II) and JS35112 (III) was 49, 41 and 49%, respectively, eigenvalues shown in Fig 4. The Jaccard’s genetic distances between resistant and susceptible parents was 0.49, 1.24 and 1.13 for cross JS31411 (I), JS34212 (II) and JS35112 (III) respectively, showing that the resistant parent I0349 was comparatively more distant than the resistant parent I0322 to the corresponding susceptible parents.

### Introgression

**Fig 5:**
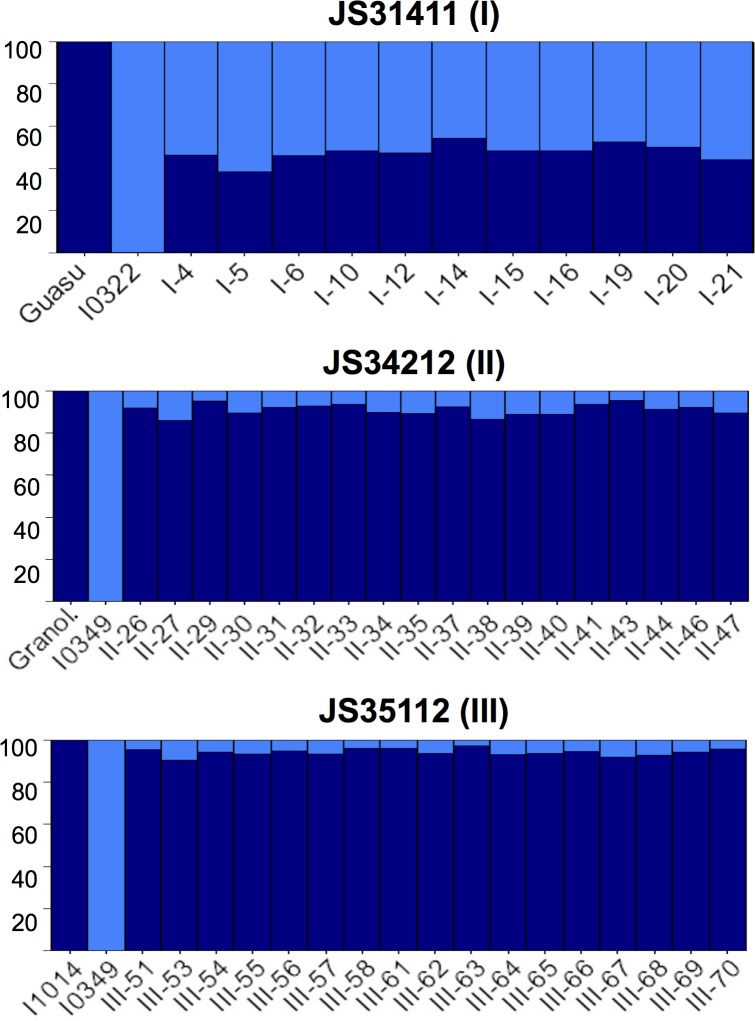
Percentage of parent-specific alleles (PSA) contributed to each of the progeny in three crosses between smut susceptible and resistant peanut plants. Dark blue: corresponds to alleles contributed by the susceptible parent, Light-blue: corresponds to alleles from the resistant parent in each cross.

Allele contributions from the parental lines followed different patterns in the three crosses analyzed. Results showed that the number of PSA from the susceptible (P1) and resistant (P2) parents was similar (16 and 18) in cross JS31411 (I) (Fig 5, Table 4). However, in crosses JS34212 (II) and JS35112 (III) the number of PSA derived from the resistant parent (I0349) was approximately one order of magnitude lower (6 and 4 for crosses JS34212 (II) and JS35112 (III), respectively) than those from the susceptible parents (61 and 71, for crosses JS34212 (II) and JS35112 (III), respectively) (Table 4). BLAST analysis of the sequences containing SSRs and InDels against the cultivated peanut v. Tifrunner (*A*. *hypogaea*) revealed several genomic regions having been introgressed from the resistant parents in all three crosses. The introgressed fragments were broadly distributed across 15 of the 20 chromosomes, with 5 to 10 chromosomes from the resistant parents being present in the progenies (Fig 5, Table 4, S2 Table, S3 Table). Crosses JS34212 (II) and JS35112 (III) had the lowest number of alleles from the resistant parent (I0349). To graphically represent the level of recombination, PSAs from susceptible and resistant parents were plotted as a percentage of total PSAs per individual, light-blue areas represent introgression from resistant parents (Fig 5). This shows that in the first cross both parents contributed similar number of PSAs to the progeny, whereas in crosses II and III, the PSA contribution from the resistant parent was an order of magnitude smaller than the contribution from the susceptible parent, Fig 5. When tested for marker trait association only one marker (cont01277a) was significantly associated (*P* ≤ 0.01) with both incidence and DI traits in two of the three crosses, JS31411 (I) and JS35112 (III). The presence of the allele (386 and 445, respectively) was associated with increased susceptibility to pod infection, and explained between 40% and 64% of the trait variation (S4 Table).

## Discussion

This is the first report of peanut smut genetic resistance identified in peanut landraces and its introgression into elite peanut cultivars. A multi-year phenotyping of three crosses between resistant landraces and susceptible elite cultivars, combined with simple-sequence repeat (SSR) and Insertion/Deletion (InDel) genotyping provided evidence of genetic introgression from the resistant germplasm.

Reliable and repeatable phenotyping remains the key to the success of any crop improvement program whether following conventional or molecular breeding approach. In this study, the evaluation of five parental lines and 53 derived RILs in an environment with high inoculum pressure allowed the detection of several inbred lines with high degree of resistance and stability over three growing seasons. Despite the environmental changes, much warmer the first year and drier on the third, our results indicated high heritability. The genetic basis underlying smut resistance has not yet been determined. However, the average heritability (*h*^*2*^) estimates observed here show a significant genetic effect and suggests major gene effects for the trait. High heritability has been previously reported in peanut soil-borne diseases such as cylindrocladium black rot (CBR) caused by *Cylindrocladium crotalariae* [49].

The presence of transgressive segregation further suggests minor genetic effects conferring both qualitative and quantitative resistance. Transgressive segregation for disease resistance has been extensively documented in wild and cultivated peanut germplasm. Recent studies include rust [50], TSWV [51, 52], and late leaf spot [53]. From a breeding perspective, the occurrence of transgressive phenotypes in advanced generations of a self-pollinated crop such as peanut is key to the improvement of resistance.

Genotyping of the RILs and corresponding parental lines provided substantial evidence of genetic recombination in all three crosses. Factors such as small population size, segregation distortion, and/or SSR/InDel marker ascertainment bias might explain the relatively low number of PSA from the resistant parent (I0349) observed in crosses JS34212 (II) and JS35112 (III). Overall similar level of transferability, 36% (134 out of 373) was observed when the set of SSR/InDel markers used here was tested on 20 wild peanut species of section Arachis, Erectoides, Heteranthae, Procumbentes and Rhizomatosae [31]. Parent specific alleles (PSA) from microsatellites have been used to demonstrate introgression in crosses between *Triticum aestivum* × *Aegilops speltoides* [54]. In this study, out of 84 markers that showed PSAs, only seven showed evidence of introgression [54]. Evaluating presence or absence of alleles/amplicons in microsatellites has been used in peanut crosses with synthetic amphidiploids to demonstrate introgression of resistant genes [55].

This study further demonstrates the benefits of landraces as a source and a pathway to broaden the genetic base for smut resistance in elite cultivars. With a higher marker density, the advanced inbred lines used in this study are an excellent genetic material for future marker-trait associations.

## Supporting information

S1 Table Smut disease incidence (%) and disease index values with mean and standard deviation (SD) for the parental lines (P1, P2) and three generations of the JS31411, JS34212, and JS35112 RILs. (XLSX)

S2 Table Genotype data of parents (P1, P2) and progeny (I, II, III) in three crosses between peanut smut resistant and susceptible germplasm, using 47 and 52 markers (SSR and InDel). (XLSX)

S3 Table SSR/InDel marker sequences position based on the *A*. *duranensis* and *A*. *ipaënsis* genome assemblies. Numbers in parentheses indicate the corresponding references as described in Table 3.
(XLSX)

S4 Table Significant marker-trait association identified in the progeny of two crosses for incidence and disease index (DI) traits. (XLSX)

S1 Fig Average monthly weather records for the peanut area of Cordoba as defined in [7]. Data were extracted from the yearly report of Bolsa de Cereales de Cordoba [27–29]. Top: average monthly maximum and minimum temperatures for the peanut area during three crop cycles (2014/15; 2015/16 and 2016/17). Bottom: average monthly precipitation for the same three crop cycles.
(TIFF)

S2 Fig Neighbor joining tree of genetic distance among parents and progeny of three crosses: JS31411 (I), JS34212 (II), and JS35112 (III).
(TIFF)
