# Supplementary figures and images for: Introgression of peanut smut resistance from landraces to elite peanut cultivars (Arachis hypogaea L.)
Source: PLoS One. 2019 Feb 8;14(2):e0211920. doi: 10.1371/journal.pone.0211920 (PMC6368304; doi:10.1371/journal.pone.0211920)

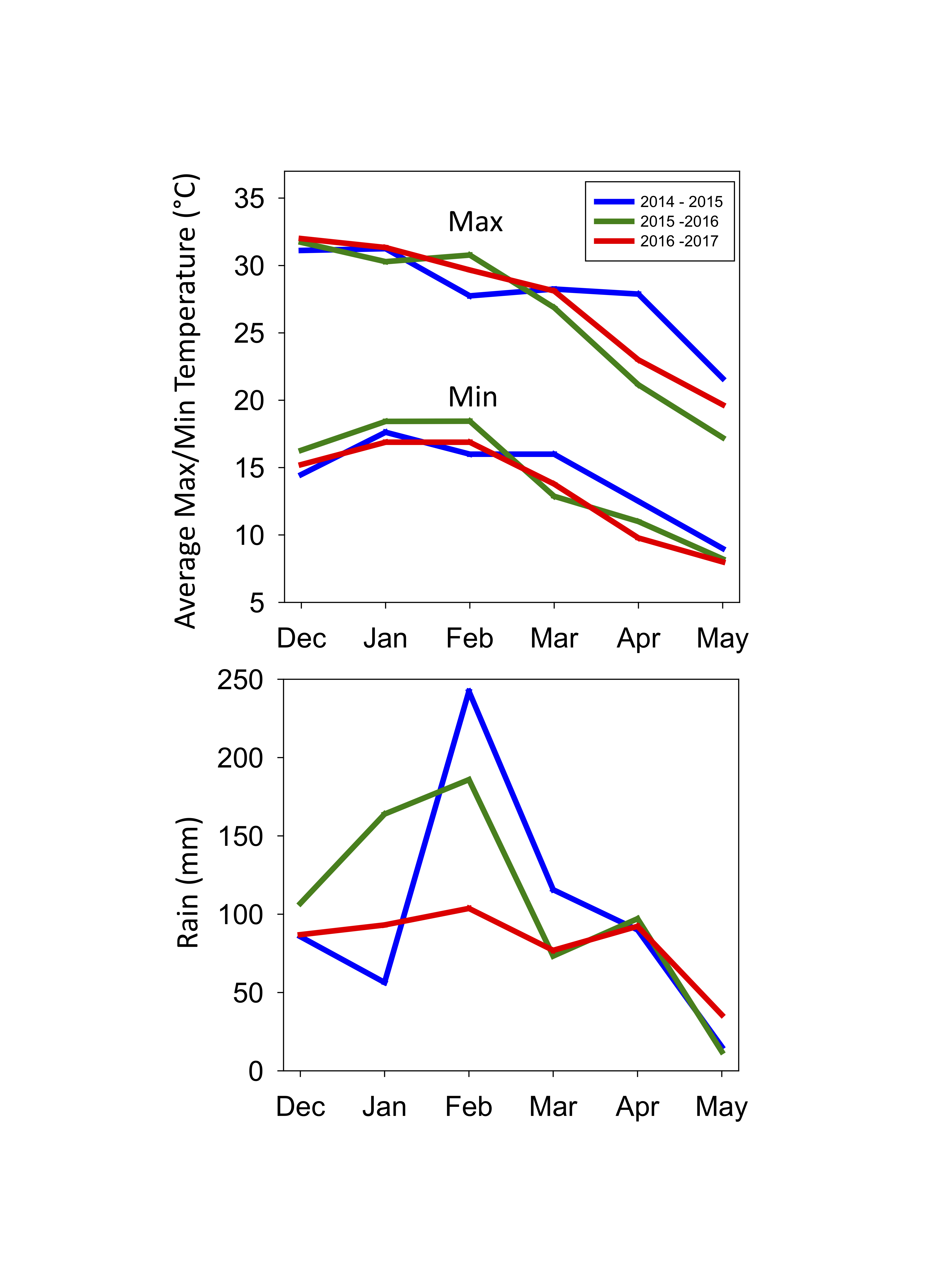

Supplement: S1 Fig — Data were extracted from the yearly report of Bolsa de Cereales de Cordoba [27–29]. Top: average monthly maximum and minimum temperatures for the peanut area during three crop cycles (2014/15; 2015/16 and 2016/17). Bottom: average monthly precipitation for the same three crop cycles. (TIFF) [file pone.0211920.s005.tiff]

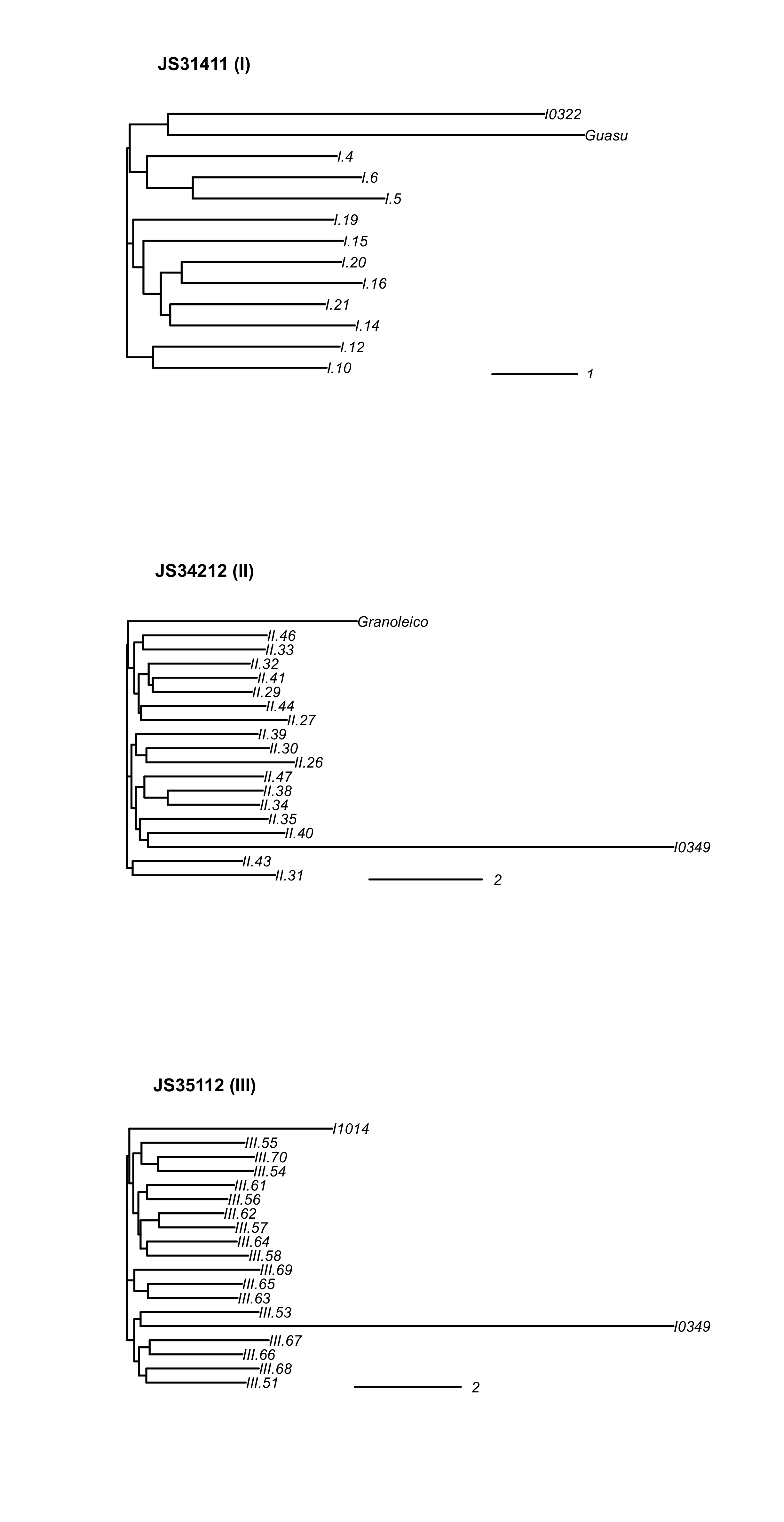

Supplement: S2 Fig — Neighbor joining tree of genetic distance among parents and progeny of three crosses: JS31411 (I), JS34212 (II), and JS35112 (III). (TIFF) [file pone.0211920.s006.tiff]
